# Traumatic Displacement of Maxillary Permanent Canine into the Vestibule of the Mouth

**DOI:** 10.1155/2015/360160

**Published:** 2015-04-27

**Authors:** Masayasu Iwase, Michiko Ito, Hanon Katayama, Hiroaki Nishijima, Hirokazu Shimotori, Airi Fukuoka, Yoko Tanaka

**Affiliations:** ^1^Department of Dentistry and Oral Surgery, Hakujikai Memorial General Hospital, 5-11-1 Shikahama, Adachi-ku, Tokyo 123-0864, Japan; ^2^Department of Oral and Maxillofacial Surgery, School of Dental Medicine, Tsurumi University, 2-1-3 Tsurumi, Tsurumi-ku, Yokohama, Kanagawa 230-8501, Japan; ^3^Division of Community Based Comprehensive Dentistry, Department of Special Needs Dentistry, School of Dentistry, Showa University, 2-1-1 Kitasenzoku, Ota-ku, Tokyo 145-8515, Japan; ^4^Department of Dentistry and Oral Surgery, Jinkokai Hospital, 3-8-11 Nakamachi, Atsugi, Kanagawa 243-0018, Japan

## Abstract

Dentoalveolar injuries are common and are caused by many factors. Dental trauma requires special consideration when a missing tooth or tooth fracture accompanies soft tissue laceration. A tooth or its fragment occasionally penetrates into soft tissue and may cause severe complications. This report presents a case of delayed diagnosis and management of a displaced tooth in the vestibule of the mouth following dentoalveolar injury. This report suggests that radiography can lead to an early diagnosis and surgical removal of an embedded tooth in the soft tissue.

## 1. Introduction

Dental trauma can result in a number of different injury types involving teeth and their supporting structures. Six types of luxation and seven types of tooth fracture have been described [1]. The frequency of lateral luxation and avulsion of teeth leading to a traumatic dental injury increases with age, while intrusion decreases with age [1]. A tooth or its fragment may displace anteriorly, posteriorly, or vertically according to the impact energy and direction of the causal agent, as well as the location of the injury and the support structure of the involved tooth. Most dentoalveolar fractures are in front of the maxilla [2]. There have been many reports of tooth fragments embedded in soft tissue accompanying a tooth fracture [3–5], but this case did not involve a tooth fracture. Furthermore, displacement of teeth most often involves the central and lateral incisors, while the canines are rarely involved [2]. Cases involving displacement of a tooth or its fragment into soft tissue resulting in dentoalveolar injury have been reported in the tongue [3, 4], lips [5, 6], and nasal cavity [2, 7] but are extremely rare in the vestibule of the mouth. When dental physicians encounter a tooth or its fragment accompanying soft tissue swelling and laceration subsequent to a dentoalveolar injury, they should pay attention to possible displacement of the tooth or its fragment into the soft tissue. Therefore, previous studies have emphasized that dental physicians should perform a clinical examination of the laceration with proper radiography in cases of dentoalveolar injury [2, 5, 6].

This paper reports a case of dentoalveolar injury in which a canine was embedded in the vestibule of the mouth and surgically removed from the soft tissue.

## 2. Case Presentation

A 46-year-old female was referred to the Department of Dentistry and Oral Surgery of Hakujikai Memorial General Hospital for clinical examination of the left maxilla with spontaneous pain. The patient had sustained an injury to the lower face 12 days earlier. She promptly consulted a neighboring emergency hospital because of laceration of the lower lip and gingiva of the maxilla. Thereafter, she was treated with suture of the lower lip under local anesthesia by a general surgeon and was instructed to put pressure on the bleeding gingiva with gauze. In addition, she lost the left maxillary lateral incisor and canine due to trauma. She could confirm the existence of one of the teeth, but the existence of the other tooth was unclear. No treatment or examination was provided for the missing teeth. She received prescriptions for antibiotics and analgesics and returned home. The bleeding easily stopped afterwards. However, because of swelling and pain of the left maxilla, the patient consulted our hospital.

**Figure 1:**
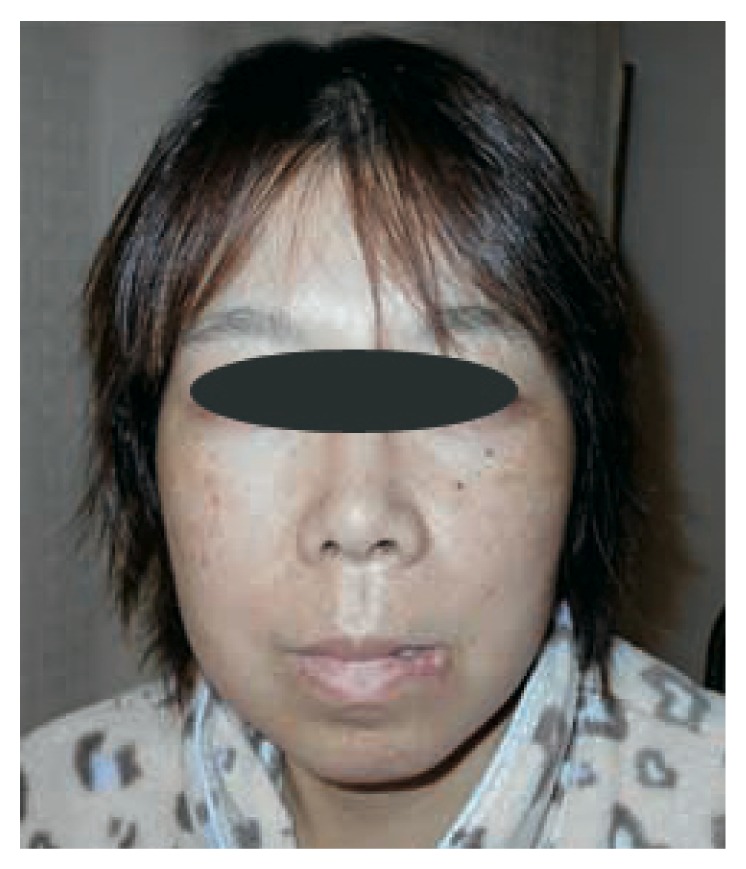
Extraoral view at first medical examination of lacerative scar in the lower lip and swelling in the nasolabial sulcus.

**Figure 2:**
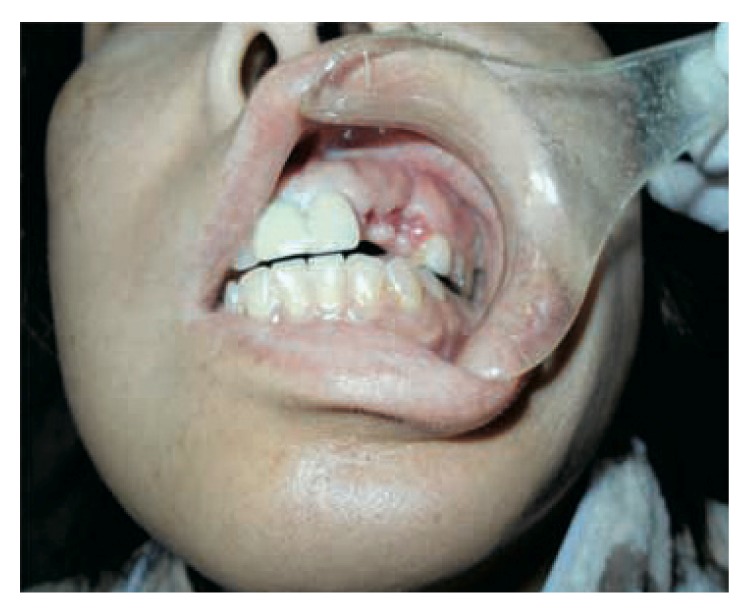
Intraoral view at first medical examination of lacerative scar of the gingiva in the left maxilla.

**Figure 3:**
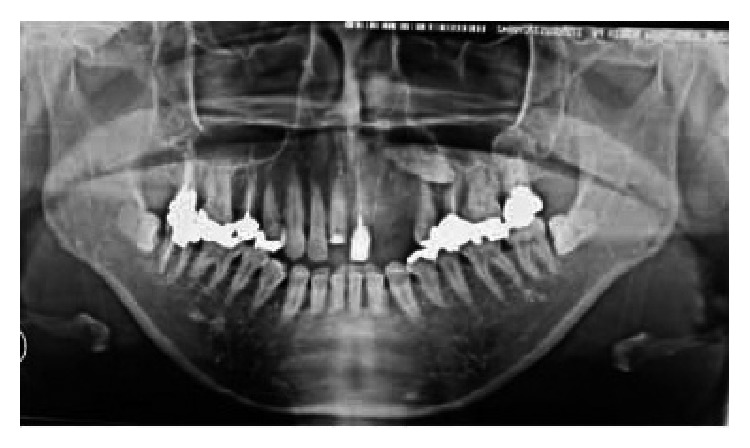
Panoramic radiograph showing horizontally embedded canine of the left maxilla.

**Figure 4:**
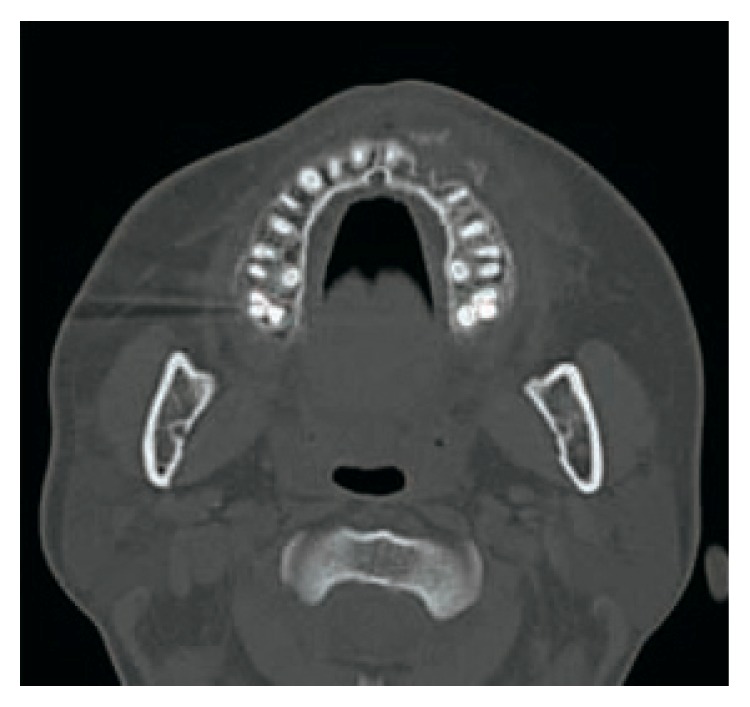
Axial CT showing fracture of alveolar bone in the lateral incisor and canine.

**Figure 5:**
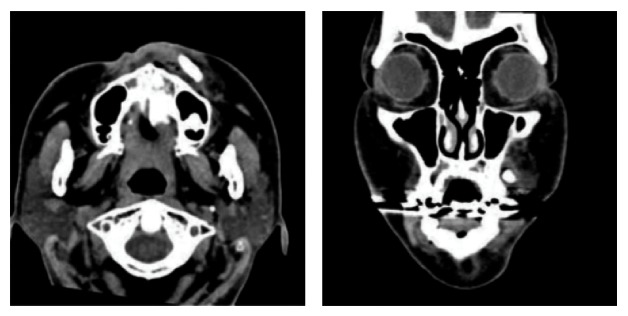
Coronal and axial CT showing embedded canine into soft tissue of the vestibule of the mouth.

**Figure 6:**
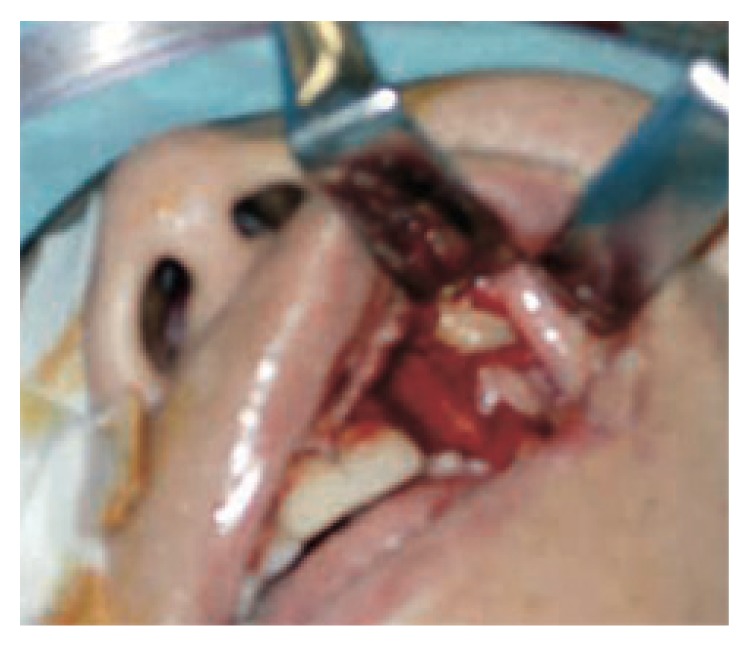
Intraoral view during operation showing exposure of embedded canine into soft tissue of the vestibule of the mouth.

**Figure 7:**
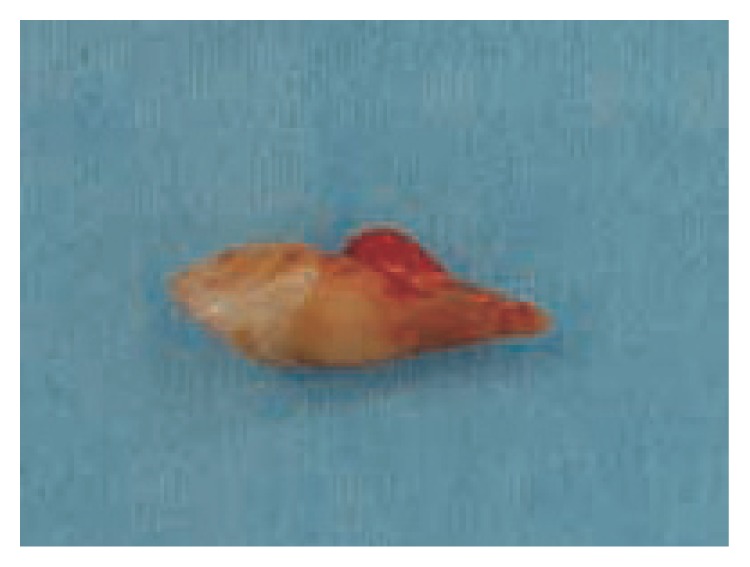
Identified and removed permanent canine.

Her chief complaint at the time of the first medical examination was swelling and oppressive pain of the left maxilla (Figure 1). Intraoral view confirmed swelling of the oral vestibule mucosa in the first premolar region (Figure 2). Panoramic radiography showed a horizontal embedded canine (Figure 3). CT scan showed fracture of the alveolar bone in parts of the lateral incisor and canine (Figure 4). Based on these findings, replantation of the canine was not an option. Furthermore, CT scan showed that the canine was embedded in the vestibule soft tissue (Figure 5). We decided to perform surgical excision of the embedded canine under local anesthesia (3.6 cc lidocaine in 2% solution with 1 : 80,000 adrenaline) together with intravenous sedation with midazolam (3 mg/body). An incision was then made in the lacerated gingiva, and the embedded canine was removed surgically. Fibrous tissue and fragments of alveolar bone surrounding the embedded canine were also curetted (Figures 6 and 7). The incision was sutured with 4.0 silk suture threads. Systemic antibiotic (1 g flomoxef sodium, twice a day for three days) was intravenously administered to the patient. Analgesic (tramadol hydrochloride/acetaminophen, 2 tablets for pain) was also prescribed to the patient. The wound healed favorably and sutures were removed on the seventh postoperative day, and the patient was discharged from the hospital.

## 3. Discussion

The recognition and identification of an embedded tooth or its fragment are important because continuous movement and contraction of the muscles may dislocate the foreign bodies. Moreover, oral bacteria flora can infect the wound and deep tissues. Failure to remove an embedded tooth or its fragment in the soft tissue may result in persistent chronic infection, pus discharge, or disfiguring fibrosis [8]. Previous reports have emphasized that radiography, including CT, should be a routine diagnostic procedure in all cases with associated missing anatomical structures in the oral and maxillofacial region [2, 5]. Involving dental professionals in the initial assessment of dentoalveolar injury in emergency rooms in hospitals is important in order to identify how many teeth might be missing after dentoalveolar injury.

This case report demonstrates the importance of an accurate patient history, physical examination, and radiographic evaluation of such a patient. When dentoalveolar injury occurs, both hard and soft tissue structures must be examined carefully for evidence of an embedded tooth.
